# Assessing the Within-Person Variability of Internal and External Sexual Consent

**DOI:** 10.1080/00224499.2021.1913567

**Published:** 2021-04-30

**Authors:** Malachi Willis, Kristen N. Jozkowski, Ana J. Bridges, Jennifer C. Veilleux, Robert E. Davis

**Affiliations:** a Institute of Health and Wellbeing, MRC/CSO Social and Public Health Sciences Unit, University of Glasgow; b Department of Applied Health Science and Kinsey Institute, Indiana University; c Department of Psychological Science, University of Arkansas; d Department of Health, Human Performance, and Recreation, University of Arkansas

## Abstract

Sexual consent is often conceptualized as an internal willingness to engage in sexual activity, which can be communicated externally to a sexual partner. Internal sexual consent comprises feelings of physical response, safety/comfort, arousal, agreement/want, and readiness; external sexual consent includes communication cues that may be explicit or implicit and verbal or nonverbal. Most previous research on sexual consent has focused on between-person differences; little attention has been devoted to examining the within-person variation of sexual consent across time. We conducted a 28-day experience sampling methodology (ESM) study with a sample of adults (*N* = 113) to assess fluctuations in internal and external sexual consent across a given person’s sexual events. We found that more than 50% and up to 80% of the variance in sexual consent scores could be accounted for by within-person variability. The type of sexual behavior participants engaged in during a sexual event predicted their internal and external consent. Further, internal consent feelings predicted external consent communication. Overall, our findings provided initial evidence regarding the extent that situational contexts are relevant for sexual consent. ESM study designs may be used to further investigate the potential contextual, intrapersonal, and interpersonal factors associated with internal and external sexual consent.

## Introduction

In the academic literature, sexual consent has been conceptualized as an internal willingness to engage in partnered sexual activity; this willingness may be expressed externally (Hickman & Muehlenhard, 1999; Jozkowski, Sanders et al., 2014). How people experience and communicate sexual consent can vary between people based on individual differences (e.g., gender; Jozkowski & Peterson, 2013) and contexts (e.g., type of sexual behavior; Marcantonio et al., 2018; Willis, Hunt et al., 2019). However, very little is known about the fluctuations of sexual consent across a given person’s sexual experiences. One approach ideally suited for investigating the potential within-person variability of sexual consent is experience sampling methodology (ESM). In the present study, we used ESM to assess the extent that internal consent feelings and external consent communication vary from experience to experience.

### Sexual Consent

Despite affirmative consent initiatives touting explicit and verbal sexual consent communication as simple and unambiguous (Beres, 2014; Curtis & Burnett, 2017; Willis & Jozkowski, 2018a), extant research suggests that sexual consent is complex and contextual. For instance, even though participants in Righi et al.’s (2019) qualitative study defined sexual consent as a verbal “yes” to engage in sexual activity, they suggested that an array of strategies – verbal and nonverbal – are used to communicate consent in actual sexual encounters. Further evidencing this discrepancy between affirmative consent initiatives and people’s lived experiences, Orchowski et al. (2020) found that some people still conceptualize consent as the absence of a “no” and not the presence of a “yes.” Given these shortcomings of attempts to portray sexual consent as unidimensional, Harris (2018) warned of the risks associated with conceptualizing sexual consent in ways that align with two key myths about communication: (1) discourse merely mirrors reality and (2) local discourse is disconnected from social/historical context.

First, sexual consent is not as simple as “yes” meaning “yes” and “no” meaning “no.” Rather, people are much more diverse in the ways they communicate their willingness to engage in sexual activity with another person (Orchowski et al., 2020; Righi et al., 2019). Despite the intricacy with which consent may be expressed, researchers suggest that people are deft communicators capable of skillfully navigating sexual situations by using and interpreting nuanced cues (Beres, 2010; Harris, 2018). Further indicating that sexual consent is not simply “yes” versus “no,” internal feelings of sexual consent may be conceptualized continuously, indicating that there is variation in people’s willingness even across partnered sexual encounters that are labeled as consensual (Jozkowski, Sanders et al., 2014). Indeed, sexual consent is an ongoing process (Harris, 2018; Humphreys, 2004; Jozkowski & Willis, 2020). For these reasons, the way people experience and communicate their sexual consent should not be represented as some stagnant reality that can be reflected by simply saying “yes” or “no.”

Second, sexual consent does not exist in a vacuum. Instead, a person’s willingness to engage in sexual activity with another person may depend heavily on the circumstances under which the encounter is to take place. For example, socially prescribed sexual scripts might inform people of the types of behaviors or types of interpersonal relationships for which sexual consent should be communicated (Humphreys, 2007). Indeed, people believe that their expectations for sexual consent should be different with committed sexual partners as opposed to casual sexual encounters (Muehlenhard et al., 2016; Orchowski et al., 2020). In addition, historical contexts like sexism and racism may influence the way people experience and communicate sexual consent (Harris, 2018). For example, based on stereotypically gendered roles, women are reinforced as gatekeepers and subsequently experience inhibited sexual agency (Hirsch et al., 2019; Pugh & Becker, 2018; Righi et al., 2019); thus, they tend to communicate their willingness to engage in sexual activity indirectly – while men are encouraged to do so directly (Curtis & Burnett, 2017; Jozkowski et al., 2017; Jozkowski & Peterson, 2013). For these reasons, context should be considered when examining sexual consent.

However, quantitative researchers have either (1) relied on methodologies that do not allow them to investigate how people feel or express their consent from experience to experience (e.g., cross-sectional study designs) or (2) collected data on sexual consent across several events (e.g., daily diary study designs) but collapsed these data and presented them as an aggregate rather than capitalizing on variation across time points. No research to our knowledge has been explicitly designed to examine the within-person variability of sexual consent and presented findings demonstrating the extent that sexual consent varies from experience to experience. Rather, much of the quantitative research on sexual consent has focused on measuring internal consent feelings or external consent communication and assessing between-person differences related to these constructs.

### Internal and External Sexual Consent

Acknowledging that consent should be conceptualized as complex and contextual – and thus able to vary from behavior to behavior, partner to partner, and situation to situation, Willis and Jozkowski (2019) defined sexual consent as one’s “willingness to engage in a particular sexual behavior with a particular person within a particular context” (p. 1723). This definition maintains that sexual consent is an internal experience – one that is distinct from, but may be related to, sexual desire (Peterson & Muehlenhard, 2007). To assess the variety of feelings associated with an internal conceptualization of sexual consent, one research team asked participants to write about the feelings they associated with being willing to engage in sexual activity (Jozkowski, Sanders et al., 2014). These researchers identified and validated five feelings that reflect internal consent (i.e., physical response, safety/comfort, arousal, agreement/want, and readiness). Alone, each feeling does not unilaterally or comprehensively represent willingness to engage in sexual activity. Thus, whether somebody is willing to engage in a particular behavior with a particular person within a particular context depends on a multidimensional process of internal feelings.

Because people cannot automatically know the feelings of others when they engage in partnered sexual activity, sexual consent should not only be conceptualized as an internal experience (Jozkowski, Sanders et al., 2014; Muehlenhard et al., 2016). Rather, sexual partners typically communicate their consent. Active consent communication refers to anything people do to indicate their willingness to engage in sexual activity and is diverse in practice; cues can be explicit or implicit and verbal or nonverbal (Beres, 2010; Orchowski et al., 2020; Righi et al., 2019). These types of active communication are independent – explicit cues might be verbal or nonverbal, and similarly verbal cues might be explicit or implicit (Hickman & Muehlenhard, 1999; Jozkowski et al., 2019; Willis et al., 2020). According to Harris (2018), being a more effective communicator does not require a single standard for how explicit or implicit one might be; rather, an awareness of the range of possible styles is needed to make informed decisions about how to address the ambiguities of interaction. Thus, people communicate their internal feelings of consent to sexual partners in diverse ways.

When developing measures of internal and external sexual consent, Jozkowski, Sanders et al. (2014) found evidence that internal feelings aligned with external indicators. Specifically, each type of active consent communication was positively correlated with each type of internal consent feeling; however, passive consent cues (e.g., communicating willingness by not resisting) were not associated with any of the internal consent feelings. The correlations between active consent communication and consent feelings were recently replicated (Walsh et al., 2019). Though significant, these associations were weak to moderate, suggesting that internal consent feelings and external consent communication are separate constructs that uniquely contribute to an overall conceptualization of sexual consent.

Further investigating the nature of the associations between internal and external sexual consent, Willis, Blunt-Vinti et al. (2019) proposed a model whereby internal consent feelings predicted the consent communication cues participants reported using – based on previous evidence that sexual cognitions tend to precede sexual behaviors (e.g., O’Sullivan & Brooks-Gunn, 2005). Their data supported this model and further corroborated findings that passive consent cues do not reliably reflect internal feelings of consent, which were instead more closely aligned with actions or words (Willis, Blunt-Vinti et al., 2019).

### Variability of Sexual Consent

Most of the previous quantitative study designs assessing the nuances of sexual consent have investigated the between-person variability of internal consent feelings and external consent communication. For example, sexual consent can vary by gender, age, race/ethnicity, and sexual precedent. First, women are generally less direct and less verbal in their consent communication (Jozkowski & Peterson, 2013); extant literature regarding internal consent indicates that gender differences may depend on the feeling in question (Jozkowski, Sanders et al., 2014). Second, people aged 18–25 in one study reported higher internal consent scores compared with those who were older than 45 (Willis, Blunt-Vinti et al., 2019). Third, racial/ethnic minorities might be less explicit and verbal in their consent cues than White participants (Walsh et al., 2019). Fourth, people who have a more established sexual history with their partner tend to rely relatively more on active communication cues to infer sexual consent (Willis & Jozkowski, 2019). In sum, the examination of individual differences related to sexual consent has prevailed in the empirical literature.

However, little is known regarding quantitative assessments of the within-person variability of internal or external sexual consent. Previous studies on how sexual consent varies by context between people provide initial evidence that a person’s consent can depend on the situation. For example, researchers have consistently shown that sexual consent can vary by type of sexual behavior (Hall, 1998; Marcantonio et al., 2018; Willis, Hunt et al., 2019). Indeed, there is a sexual script that consent does not need to be actively communicated for lower-order behaviors according to sexual hierarchies established by previous research (e.g., Sanders et al., 2010). For example, the proportion of students who believed that explicit consent is necessary increased as the perceived level of intimacy of the sexual behavior increased (Humphreys, 2007; Jozkowski, Peterson et al., 2014). People’s consent communication behaviors reflect this belief. In a recent study, explicit verbal cues were reported with increasing frequency for the following sexual behaviors: genital touching (22.0%), oral sex (43.5%), vaginal-penile sex (57.4%), and anal sex (80.1%) (Willis, Hunt et al., 2019). While contextual factors like type of sexual behavior give insight into the potential within-person variability of sexual consent, they have typically been assessed cross-sectionally in previous studies. As such, most conclusions drawn from extant research on the contextual nuances of sexual consent are based on between-person differences at a single moment in time – rather than within-person differences across time – and are thus unable to discuss potential fluctuations in sexual consent across a given person’s experiences.

To assess within-person variability, a few research teams have asked participants about sexual consent multiple times over a study period (e.g., using daily diaries). For example, Willis and Jozkowski (2019) asked participants every day for 30 days whether they had engaged in sexual activity that day. On days that participants had engaged in partnered sexual activity, they rated whether the sexual behavior was consensual and described how they determined that rating via open-ended text responses. Willis and Jozkowski (2019) found that whether sexual consent was reportedly communicated varied not only between people but also within people across events. For example, on some days a person might have relied on active communication to interpret sexual consent with their sexual partner (e.g., “She asked if I wanted to have sex”); however, on other days, that same person may have reported they assumed consent without using communication cues (e.g., “It just happened;” Willis & Jozkowski, 2019, p. 1729). These open-ended daily diary data suggested that sexual consent is not stable from one partnered sexual event to the next within the same person. However, that study and others that have used daily diaries to collect data on constructs related to sexual consent (e.g., sexual compliance [O’Sullivan & Allgeier, 1998] and sexual initiation [Vannier & O’Sullivan, 2011]) presented the quantitative data as an aggregate; therefore, an adequate assessment of how sexual consent varies across experiences is still lacking. Willis and Jozkowski (2019) urged researchers to employ methodologies and analyses that can estimate the potential variation in sexual consent across contexts.

### Experience Sampling Methodology

The need to design studies that can capture the within-person variability of sexual consent remains. ESM, also referred to as ecological momentary assessment, provides a powerful approach for advancing research on sexual consent, primarily due to its ability to differentiate within- and between-person factors (Csikszentmihalyi & Larson, 2014; Myin-Germeys et al., 2018). Aggregating data across time points – as previous research on sexual consent has done – eliminates the ability to quantitatively assess within-person variability (Schwartz & Stone, 1998). Alternatively, using appropriate analytic strategies to evaluate ESM data (e.g., multilevel modeling) can provide researchers the ability to address research questions regarding experience-to-experience fluctuations in sexual consent.

In addition, ESM builds on traditional retrospective cross-sectional study designs by reducing recall bias. By collecting data in the moment (or close to it), ESM studies lessen the need for participants to recollect and reconstruct their memories – processes that are prone to biases (Csikszentmihalyi & Larson, 2014; Iida et al., 2012). Minimizing the time between events of interest (e.g., partnered sexual activity) and the participants’ reports regarding those events helps reduce the potential recall bias inherent to most retrospective self-reported data (McCallum & Peterson, 2012; Willis & Jozkowski, 2018b), which comprise much of the empirical literature on sexual consent. According to Simons et al. (2019), ESM may be particularly advantageous for assessing affective and cognitive factors (e.g., internal consent feelings) or continuous behavioral processes (e.g., sexual consent communication) in the moment, whereas discrete behaviors (e.g., condom use) might be less susceptible to recall bias.

Finally, ESM studies improve the ecological validity of their findings by asking people about their experiences in their natural environments (Csikszentmihalyi & Larson, 2014; Myin-Germeys et al., 2018). Thus, ESM is suitable for investigating everyday occurrences, such as partnered sexual activity. Data that are more ecologically valid can help researchers understand such nuances as the associations between internal consent feelings and external consent communication – experiences and behaviors that typically cannot be replicated in a laboratory setting.

### Present Study

Following recommendations by Willis and Jozkowski (2019), we used ESM to examine the within-person variability of sexual consent. Using validated measures of sexual consent that are feasible for ESM studies (Willis, Jozkowski et al., 2021), we assessed people’s internal consent feelings and external consent communication over a 28-day period. We had two specific research aims in this study.

For our first research question (RQ1), we aimed to quantify the extent that internal and external sexual consent vary across partnered sexual events within the same person because previous open-ended data have suggested there may be non-trivial within-person variability regarding sexual consent (Willis & Jozkowski, 2019). For the second (RQ2), we used multilevel models to account for this within-person variability and examine associations that have been reported in previous cross-sectional studies. Specifically, we assessed several between-person predictors of internal and external sexual consent that have been identified as potentially relevant correlates: gender, age, race/ethnicity, and relationship length. Further, we tested the within-person effects of type of sexual behavior on internal and external sexual consent across sexual experiences as well as whether internal consent feelings positively predicted active consent communication cues at the event level.

## Method

### Participants

We recruited participants via social media (e.g., study recruitment pages on Reddit and Facebook) and a campus-wide e-newsletter at a university in the southern United States to complete an eligibility screener. To be eligible, participants had to be at least 18 years old, have daily access to a device supported by iOS (e.g., iPhone) or Android (e.g., smartphone), and be sexually active. Similar to Willis and Jozkowski (2019), we defined “sexually active” as having had participated in sexual activity (e.g., passionate kissing, oral sex, vaginal sex, anal sex) on at least two days in the preceding week. In addition, screener participants who shared the same geolocation and IP address were considered to be potential sexual partners if their responses regarding sexual behaviors in the past week were similar. If eligible based on the other criteria, we only invited the first of these pairs to participate in the full study to avoid dyadic dependencies in the data.

Of the 545 people who completed the screener survey, we invited 218 (40.0%) to participate in the ESM study. Of these, 159 (72.9%) completed the baseline survey; however, 21 (7.5%) of those participants never downloaded the ESM application onto their personal devices. In sum, 138 people began this 28-day ESM study. Twenty-one (15.2%) people withdrew from the study for personal or unknown reasons. Further, because our primary research aims focused on within-person variability, we removed data from four participants (2.9%) who did not report at least two partnered sexual events during the study period. Thus, the final analytic sample for the present study comprised 113 participants – 100 (88.5%) of whom were in the United States during the study period.

On average, the participants included in the analytic sample were 29.2 years old (*SD* = 6.5), ranging from 21 to 65. In line with this younger average age, 13 (11.5%) participants were undergraduate students, 55 (48.7%) were graduate students, and 45 (39.8%) were not students. Regarding gender, 65 (57.5%) identified as women, 47 (41.6%) as men, and 1 (0.9%) as gender fluid. Regarding race/ethnicity, 80 (70.8%) participants identified as White or European American, 12 (10.6%) as Hispanic or Latin American, 11 (9.7%) as Asian or Asian American, 4 (3.5%) as Black or African American, and 6 (5.3%) as another race/ethnicity or multiple races/ethnicities. Regarding sexual orientation, 82 (72.6%) participants identified as heterosexual, 19 (16.8%) as bisexual, and 12 (10.6%) as another sexual orientation. Participants had been in a committed relationship with their current sexual partner for an average of 5.8 years (*SD *= 5.8), ranging from 0.3 to 35.3 years.

### Procedure

Interested people who clicked on the recruitment link were directed to an introductory page that provided them with information about the study and screener questions using Qualtrics survey software. Those eligible were provided a link to the baseline survey, which included the consent form for the 28-day study and sociodemographic items. This baseline survey was completed via Qualtrics survey software on a personal computer at a location of their choosing. Those who completed the baseline survey received instructions for downloading the LifeData application^1^ ^1^The LifeData application prompted participants to complete ESM surveys, time-stamped the responses, and stored the data on a secure server. The LifeData application did not record any identifying information from the participant’s personal device. (lifedatacorp.com) onto their device.

The 28-day ESM study took place from 11th April 2020 to 8th May 2020 for all participants. ESM surveys were sent to participants three times a day using a semi-random sampling scheme (i.e., random sampling within three fixed windows every day). The specific windows were 7am–9am, 1pm–3pm, and 7pm–9pm (participants’ local time). If participants engaged in partnered sexual activity since their previous survey, they responded to measures of internal and external sexual consent. If not, they responded to items on sexual interest and solo sexual behaviors (which were unrelated to the goals of the present study), resulting in surveys of similar length on both tracks – decreasing incentive to falsely report a lack of partnered sexual activity to receive a shorter ESM survey (Willis & Jozkowski, 2019).

Based on the number of ESM surveys they completed, participants received up to a 40 USD Amazon.com e-gift card for their participation. The procedure for this 28-day ESM study was approved by the university’s institutional review board.

### Measures

#### Sociodemographic Variables

We measured four sociodemographic variables that previous research has suggested may be relevant for sexual consent: gender, age, race/ethnicity, and relationship length. Gender was measured dichotomously (i.e., 0 = woman; 1 = man). Age and relationship length were measured continuously in years. Race/ethnicity was measured dichotomously (i.e., 0 = identified as a racial/ethnic minority; 1 = identified as White).

#### Type of Sexual Behavior

In each of the daily surveys, participants responded to an item that asked about recent partnered sexual activity: “Since the last beep, I engaged in the following behaviors with my partner.” Response options included passionate kissing, genital touching, oral sex, vaginal sex, and anal sex; participants were instructed to select all that applied. We created an ordinal event-level variable to capture type of sexual behavior according to previously established sexual hierarchies (i.e., 0 = passionate kissing only, 1 = genital touching but not higher behaviors, 2 = oral sex but not higher behaviors, 3 = vaginal sex but not anal sex, 4 = anal sex; Sanders et al., 2010).

#### Internal Sexual Consent

At time points that participants reported a recent partnered sexual event, they responded to five items developed and validated to measure internal sexual consent using ESM (Table 1). Response options for each of these items measuring internal sexual consent were provided on aunipolar 11-point sliding scale (“Not at all” to “Very much”). These items demonstrated good internal reliability (α = .84). Higher composite scores indicate greater feelings of internal sexual consent at a given partnered sexual event.

**Table 1.** ESM items measuring internal and external sexual consent

	Item Wording	Target Construct
Internal Sexual Consent	1. During these sexual behaviors, I felt erect/vaginally lubricated	Physical response
	2. During these sexual behaviors, I felt comfortable	Safety/comfort
	3. During these sexual behaviors, I felt turned on	Arousal
	4. During these sexual behaviors, the sexual act itself felt consensual	Agreement/want
	5. During these sexual behaviors, I felt ready	Readiness
External Sexual Consent	1. I used straightforward signals to communicate my willingness to engage in these sexual behaviors	Explicit cues
	2. I used subtle signals to communicate my willingness to engage in these sexual behaviors	Implicit cues
	3. I verbally communicated my willingness to engage in these sexual behaviors	Verbal cues
	4. I nonverbally communicated my willingness to engage in these sexual behaviors	Nonverbal cues

These items were designed to assess five types of internal consent feelings and four types of active consent communication. The operational definitions that were used to develop these items are included in Willis, Jozkowski et al. (2021). Participants were provided the operational definitions for these constructs before participating in the study.

#### External Sexual Consent

At time points that participants reported a recent partnered sexual event, they also responded to four items developed and validated to measure active consent communication using ESM (Table 1). Response options for each of these items measuring sexual consent were provided on a unidimensional 11-point sliding scale (“Not at all” to “Very much”). These items demonstrated satisfactory but not strong internal reliability (α = .61). Higher composite scores indicate greater use of active cues to communicate willingness to engage in a given partnered sexual event.

### Analysis

Regarding our initial assessment of the data, we calculated event-level statistics for the ESM measures of sexual consent as well as person-level statistics (e.g., aggregating time points). We examined both event- and person-level bivariate correlations to disaggregate within- and between-person sources of variance, respectively. Descriptive statistics and bivariate correlations were produced using SPSS 26.

To answer our specific research questions, we estimated multilevel models (described in detail below). For these models, we reported unstandardized coefficients and standard errors for each predictor variable. These parameters were evaluated at an α of .05. Regarding data-model fit, nested models with relatively smaller values for the Akaike Index Criterion (AIC) and Bayesian Index Criterion (BIC) were considered to fit the data better. We tested these multilevel models using the “lme4” package in *R* (Bates et al., 2015) and conducted a post hoc power analysis using the “EMAtools” package (Kleiman, 2017). With 113 participants, a 28-day study period, three time points per day, an estimated ICC of .5, and a completion rate of 84%, we were adequately powered (1 – *β *= .8) to detect medium effect sizes in our multilevel models (see Online Supplementary Material for the power curve associated with these study parameters).

First, to examine the extent that participants’ reports of internal and external sexual consent significantly varied within people across experiences (RQ1), we calculated intraclass correlation coefficients (ICCs), which range from 0 to 1. These ICCs were estimated using unconditional multilevel models (i.e., without predictors) that nested time points within people and indicated how much variation in participants’ reports of internal consent feelings and active consent communication could be attributed to between-person differences. Subtracting the ICCs from 1 provided the amount of variance accounted for by within-person differences.

Second, to assess predictors of internal and external sexual consent while accounting for both within- and between-person variability (RQ2), we tested conditional multilevel models that were estimated using random intercepts. For the model with internal consent feelings as the dependent variable, type of sexual behavior was an event-level predictor variable (level 1) and person-level predictor variables included gender, age, race/ethnicity, and relationship length (level 2). These same predictors were included in the model with external consent communication as the dependent variable, and the event-level score for internal consent feelings was an additional predictor (level 1). Event-level predictor variables were centered at the person-level means; as in simple regression, dependent variables were not centered. Effects were only modeled for time points in which participants reported a partnered sexual event because participants did not respond to the measures of internal or external sexual consent if they had not recently engaged in partnered sexual activity.

## Results

### Descriptive Statistics

Across the 113 participants, a total of 9492 surveys were distributed (i.e., three surveys each day for 28 days). In sum, 7969 surveys were completed; thus, the overall compliance rate was 84.0%. Participants reported a total of 1192 partnered sexual events during the study period (15.0% of completed time points). Reported partnered sexual events with any missing data were removed, resulting in an analytic sample of 1189 events.

At the person level, the mean number of partnered sexual events was 10.5 over the 28-day study period (*SD* = 7.5), ranging from 2 to 39. Across partnered sexual events, participants reported engaging in passionate kissing a total of 961 times (80.8%), genital touching 976 times (82.1%), oral sex 603 times (50.7%), vaginal sex 777 times (65.3%), and anal sex 60 times (5.0%).

For most partnered sexual events, participants endorsed relatively high levels of internal sexual consent, *M* = 8.70, *SD* = 1.42, ranging from 1.2 to 10. There was relatively more variability in how participants communicated their willingness during partnered sexual events, *M* = 5.40, *SD* = 2.10, ranging from 0 to 10. The composite scores for internal and external sexual consent were approximately normally distributed at the event- and person-level or did not have substantially non-normal distributions (Ryu, 2011).

### Bivariate Correlations

At the event level, internal and external sexual consent were significantly correlated; partnered sexual events in which participants reported greater levels of internal consent feelings were associated with greater use of active consent communication, *r* = .12, *p* < .001. Further, events that involved increasingly intimate sexual behaviors were positively associated with internal sexual consent, *r* = .36, *p* < .001, and external sexual consent, *r* = .13, *p* < .001.

When averaged across time points, participants’ composite scores for internal consent feelings were not associated with their average composite scores for external consent communication, *r* = .34, *p* = .090. Neither of the person-level composite scores for internal nor external consent were significantly associated with any of the sociodemographic characteristics of interest: gender, age, race/ethnicity, or relationship length.

Event- and person-level bivariate correlations (as well as descriptive statistics) for the individual items composing internal consent feelings (i.e., physical response, safety/comfort, arousal, agreement/want, readiness) and external consent communication (i.e., explicit, implicit, verbal, nonverbal) are provided in the Online Supplementary Material.

### RQ1: Unconditional Multilevel Models

We tested unconditional multilevel models to estimate the ICCs for internal and external sexual consent. ICCs for each item are presented in Table 2. The ICC for the internal sexual consent composite score was .449, which meant that approximately 55% of its variance across sexual experiences could be accounted for by within-person variability. ICCs for individual internal consent feelings ranged from .326 to .444. Even more of the variance (72.5%) in active consent communication across experiences could be attributed to within-person variation (ICC = .275), with ICCs for individual aspects of external sexual consent ranging from .189 to .414.

**Table 2.** Intraclass correlation coefficients for internal and external sexual consent

	ICC	95% CI	1 – ICC
Internal Sexual Consent			
Physical response	.326	[.254, .396]	.674
Safety/comfort	.444	[.372, .524]	.556
Arousal	.356	[.288, .436]	.644
Agreement/want	.402	[.331, .482]	.598
Readiness	.357	[.289, .437]	.643
External Sexual Consent			
Explicit cues	.189	[.136, .256]	.811
Implicit cues	.414	[.344, .495]	.586
Verbal cues	.233	[.175, .305]	.767
Nonverbal cues	.366	[.298, .447]	.634

Each ICC indicates the proportion of variance that can be accounted for by between-person variability. 1 – ICC indicates the proportion of variance that can be accounted for by within-person variability.

### RQ2: Conditional Multilevel Models

We tested conditional multilevel models to examine the predictors of internal and external sexual consent while accounting for the substantial within-personal variability in these outcome variables as indicated by the ICCs. Table 3 presents the fixed effects for the reduced models predicting internal consent feelings or external consent communication (see Online Supplementary Material for the fixed effects of the full conditional models).

**Table 3.** Fixed effects for the reduced multilevel models predicting internal and external sexual consent (N = 113)

	Internal Sexual Consent				External Sexual Consent			
	*β*	SE	95% CI	*p*	*β*	SE	95% CI	*p*
Intercept	7.66***	.17	[7.33, 7.99]	< .001	4.63***	.22	[4.20, 5.06]	< .001
Type of sexual behavior	.46***	.05	[.37, .55]	< .001	.32***	.09	[.15, .48]	< .001
Internal sexual consent	–	–	–	–	.11*	.06	[.00, .23]	.046

*p < .05. ***p < .001.

The conditional model predicting internal sexual consent fit the data better than the unconditional model, ΔAIC = −176.9, ΔBIC = −141.4. Because there were no significant effects of gender, age, race/ethnicity, or relationship length, we removed these person-level predictors and interpreted the effects of a more parsimonious reduced model – which also allowed us to include the participant who identified as gender fluid. The fixed effects of this reduced model explained 10.9% of the variance in internal sexual consent scores; the random effects accounted for an additional 41.8%, Conditional *R*^2^ = .537. Type of sexual behavior significantly predicted event-level internal consent feelings. Specifically, partnered sexual events that included sexual behaviors of increasing intimacy were associated with greater levels of internal consent feelings, *β* = .46, *p* < .001.

The conditional model predicting external sexual consent fit the data slightly better than the unconditional model, ΔAIC = −33.5, ΔBIC = 22.4. Because there were no significant effects of gender, age, race/ethnicity, or relationship length, we again removed these person-level predictors and interpreted the effects of a reduced model. The fixed effects of this reduced model explained 3.0% of the variance in internal sexual consent scores; the random effects accounted for an additional 28.1%, Conditional *R*^2^ = .311. Type of sexual behavior and event-level internal consent feelings significantly predicted event-level external sexual consent. First, partnered sexual events that included sexual behaviors of increasing intimacy were associated with greater levels of active consent communication, *β* = .32, *p* < .001. Second, partnered sexual events for which participants reported greater levels of internal consent were associated with greater levels of active consent communication, *β* = .11, *p* = .046.

Because the internal consistency of the items measuring external sexual consent was not strong, we tested follow-up multilevel models to individually assess the associations between internal consent feelings and the four types of active consent communication. These post hoc analyses demonstrated that greater event-level internal sexual consent significantly predicted event-level use of each type of active consent cue: explicit (*β* = .61, *p* < .001), implicit (*β* = .31, *p* < .001), verbal (*β* = .53, *p* < .001), and nonverbal (*β* = .45, *p* < .001). See Online Supplementary Material for more details regarding these models.

## Discussion

Sexual consent is complex and can vary from one context to another. Building on the cross-sectional designs of previous studies that have investigated the between-person variability of sexual consent, we used ESM to gather multiple points of data over 28 days. In doing so, we provided one of the first in-depth quantitative accounts of the within-person variability of sexual consent.

We found substantial fluctuations in people’s internal and external sexual consent across sexual events. In fact, within-person variability accounted for at least 50% and up to 80% of the variation in all five consent feelings and each of the four types of active consent communication. Because the extant body of research on sexual consent has relied on cross-sectional investigations of between-person differences, much of what seems to contribute to their experiences or communication of sexual consent remains unexplored. However, designing studies to capture experience-to-experience fluctuations – as we did in the present study – helps obtain a more comprehensive account of the nuances of sexual consent.

Using multilevel models to account for this within-person variation, we did not corroborate cross-sectional studies that have reported between-person sociodemographic variations in sexual consent. Of note, we did not find internal or external sexual consent to be associated with gender, age, race/ethnicity, or relationship length in the tested models. Despite several studies finding cross-sectional differences in sexual consent between women and men (e.g., Hirsch et al., 2019; Jozkowski, Peterson et al., 2014; Willis, Hunt et al., 2019), such gender differences, for example, should not be assumed to be stable across contexts because people seem to be more dynamic than static in their internal and external sexual consent from one partnered sexual event to the another.

That we did not corroborate previous findings regarding between-person sociodemographic variability of sexual consent may have been due to our sample comprising only people who were in committed sexual relationships.^2^ ^2^Although we did not require participants to be in a committed sexual relationship at the time of the study, our inclusion criterion regarding sexual activity in the previous week seemed to restrict the sample to those with committed sexual partners when we collected data in April 2020 due to pandemic-related social distancing measures. Indeed, how people conceptualize sexual consent can vary based on relationship status (Humphreys & Brousseau, 2010; Orchowski et al., 2020; Righi et al., 2019), as can the ways they experience and communicate willingness to engage in sexual activity (Jozkowski, Sanders et al., 2014; Marcantonio et al., 2018; Walsh et al., 2019). Because previous studies on the between-person variability of sexual consent collected data from samples that were much more heterogeneous than the present study regarding relational context, comparisons of between-person effects across studies must be done with caution. Future ESM studies should be conducted with people in casual or novel sexual relationships to more comprehensively understand potential between-person differences in sexual consent.

Still, our findings suggest that the situational context of a sexual encounter may be more important than individual differences in how people – at least those in committed relationships – experience and communicate sexual consent. For example, we found that the type of sexual behavior a person engaged in during a given partnered sexual event was a strong predictor of people’s experiences and communication of sexual consent. In line with previous research (Humphreys, 2007; Willis, Hunt et al., 2019), we found that people reported greater levels of willingness and active consent communication during events that involved increasingly intimate types of sexual behavior. That type of behavior was associated with internal consent feelings might be due to greater levels of willingness being required for greater intimacy. In other words, partnered people might not need to feel as physically responsive, safe, aroused, in agreement, or ready to participate in consensual passionate kissing compared with consensual vaginal sex. Further, the association between type of behavior and active consent communication may reflect partnered people’s belief that consent does not necessarily need to be communicated (and thus may be assumed) for lower-order sexual behaviors (Humphreys, 2007; Muehlenhard et al., 2016). Because people’s experiences with sexual consent are complex and vary across contexts like type of sexual behavior, efforts to promote sexual consent as unidimensional may falter.

Our multilevel models also provided evidence that internal consent feelings predicted external consent communication across partnered sexual events, which supports extant cross-sectional research (Jozkowski, Sanders et al., 2014; Walsh et al., 2019; Willis, Marcantonio et al., 2021). Specifically, during sexual encounters in which participants more strongly experienced feelings associated with internal sexual consent, they were more active in communicating their willingness via cues that were explicit, implicit, verbal, or nonverbal. Previous research has similarly suggested that each of these consent cues reflects greater feelings of willingness but that passive consent communication (e.g., not resisting or not saying “no”) is not associated with internal consent feelings (Willis, Blunt-Vinti et al., 2019). Therefore, sexual consent education initiatives should encourage people to be familiar with and able to use diverse active consent communication cues to infer whether their partner is ready and willing to engage in sexual activity; relying solely on an explicit and verbal “yes” does not necessarily represent humans’ capacity to navigate complex social situations (Harris, 2018). Finally, that partnered people were on average more active in their communication if they felt greater levels of willingness at a given moment in time and within a given context indicates that sexual consent should not be assumed even within committed relationships.

### Future Directions

Because more than half the variance in sexual consent was at the within-person level in this sample, understanding momentary sexual consent remains an important research aim. A prominent challenge of sexual consent research going forward will be identifying the characteristics (e.g., trait, relational, situational) that contribute to this observed within-person variability of sexual consent from day to day – a challenge for which ESM study designs are ideally suited. Our work on internal and external sexual consent should also be complemented by studies on the within-person variability of sexual consent perceptions, which is a third component of sexual consent that was not assessed in the present study (Muehlenhard et al., 2016). Collecting data from sexual dyads using ESM would be an effective way to examine sexual consent as an interactive process, which may also vary from one context to the next.

Explanatory models or prevention efforts that fail to consider and emphasize the contextual nature of people’s willingness to engage in sexual activity seem to be missing much of the variability in sexual consent communication as a target behavior (Simons et al., 2019). Future studies should consider what contexts are associated with actively communicating consent, which in turn can reflect greater internal consent feelings (Jozkowski, Sanders et al., 2014; Willis, Marcantonio et al., 2021). Study designs that incorporate the variation of sexual consent communication within people across time will be able to investigate why a person might rely more on certain cues on some occasions but not others. Understanding the contextual variables that predict a person’s consent communication for a given sexual encounter could help improve the effectiveness of prevention and education programs designed to increase people’s reliance on active consent cues to communicate their willingness or infer that of others.

There are many potential constructs that may be relevant to sexual consent in the moment and, if supported by future research, would be worth considering for prevention or education efforts. Daily intrapersonal or interpersonal characteristics, such as a person’s mood or relationship satisfaction, might affect a person’s experience of sexual consent that day. Further, there is cross-sectional evidence that the situational contexts of a partnered sexual event can influence people’s feelings or communication of willingness – contexts like alcohol consumption (Drouin et al., 2019; Jozkowski & Wiersma, 2015). In addition to understanding the antecedents of internal and external sexual consent, future studies might also investigate the potential intrapersonal or interpersonal consequences of partnered sexual events that are associated with relatively higher or lower levels of sexual consent feelings. Researchers have posited that positive experiences regarding sexual consent may lead to increased sexual pleasure or general sexual well-being (Marcantonio et al., 2020). This would be a worthy pursuit for further investigation.

Finally, these measures of internal and external sexual consent are not conducive to a dichotomous conceptualization of whether a given sexual event was consensual; indeed, they were created from scales that were intended to measure variations within the context of consensual sexual activity (Jozkowski, Sanders et al., 2014). Though sexual assault is inextricably intertwined with sexual consent, researchers have argued that it is meaningfully different to explicitly investigate consensual sexual activity – as opposed to nonconsensual sexual activity (Humphreys, 2007; Jozkowski & Peterson, 2013). As such, any claims regarding nonconsensual sexual activity made from the present data should be made tentatively. Future work is needed to investigate how people experience, communicate, and perceive constructs like unwillingness and ambivalence – all of which will likely inform our broader understanding of sexual consent. Researchers can also build on the present study by considering people’s willingness or unwillingness to engage in sexual activity even at time points for which partnered sexual activity ultimately did not occur (e.g., consensual refusals); this approach will similarly contribute to conceptualizations of sexual consent processes. By expanding studies to consider broader constructs related to sexual consent, researchers could potentially assess how a person’s willingness may even fluctuate within a partnered sexual event, which our methodology was unable to capture.

### Strengths and Limitations

The primary strength of the present study was its use of ESM to assess the within-person variability of sexual consent. Study designs that employ ESM can overcome the limitations of previous research on sexual consent in at least three ways. First, while previous studies have collected multiple data points from participants regarding their consent to sexual activity (Vannier & O’Sullivan, 2011; Willis & Jozkowski, 2019), little to no work has investigated experience-to-experience fluctuations in sexual consent using sophisticated statistical analyses (e.g., multilevel modeling). An additional strength of our study design was that it reduced recall biases inherent in self-reported retrospective sexual behavior data (Graham et al., 2003; Willis & Jozkowski, 2018b); however, other biases (e.g., social desirability) remain a concern. Finally, by asking participants to complete daily surveys in their typical settings, we likely improved the ecological validity of our findings.

A persistent limitation of ESM studies is the lack of validated measures (Ebner-Priemer & Trull, 2009). Because ESM researchers must consider feasibility and participant fatigue when designing their studies, they typically adopt a single item or a few items from scales that were developed for traditional cross-sectional retrospective survey designs (Myin-Germeys et al., 2018; Van Berkel et al., 2017). For example, they might select the item(s) with the largest factor loading(s) (Fisher & To, 2012). Thus, another strength of the present study was our use of measures that underwent a rigorous development process to ensure their validity (i.e., face, content, convergent, and divergent) and reliability (Willis, Jozkowski et al., 2021). Still, these measures may be limited in that (1) although we asked participants at the beginning of the study to only reference sexual activity with their primary partner, we were unable to determine with certainty whether they only reported sexual behavior with that same person during the study period and (2) even though we provided participants with operational definitions, they had to engage in a degree of meta-cognition to identify their sexual consent communication as explicit or implicit. We intentionally did not asked behavior-specific questions about external sexual consent because communication cues are diverse and attempting to condense them into measures brief enough for ESM studies would not be feasible; however, Jozkowski, Sanders et al.’s (2014) 18-item External Consent Scale assesses specific behaviors and may be used in cross-sectional surveys.

The present study’s sample size (*N* = 113) was a strength and a limitation. In Van Berkel et al.’s (2017) methodological review of ESM studies that used mobile devices, the mean number of participants was 53, with half of the studies having 19 or fewer participants. Therefore, our sample size was relatively high for this type of study design; however, generalizing findings to the larger population of sexually active adults should be done with caution. That said, the present study’s sample was more heterogeneous regarding age, gender, and sexual orientation than most previous cross-sectional studies on sexual consent (Willis, Blunt-Vinti et al., 2019).

Because higher compliance rates indicate a more comprehensive assessment of the constructs of interest, another strength of the present study was its 84.0% compliance rate – more than one standard deviation above the average compliance rate of 69.6% across ESM studies included in Van Berkel et al.’s (2017) methodological review. In that review, studies that provided compensation based on how many surveys participants completed had the highest compliance rates; our study supported this association.

A further consideration and potential limitation of ESM studies is their proclivity to reactivity, which occurs when experiences or behaviors are affected by the act of assessing them. Self-monitoring has been used as an effective strategy for changing behavior – a desired outcome for many interventions (Korotitsch & Nelson-Gray, 1999). However, such changes in experience or behavior are not welcomed in research designed to examine naturally occurring phenomena (Simons et al., 2019). Future research should investigate the extent that reporting internal and external sexual consent over a study period might influence participants’ partnered sexual events and consider ways to reduce reactivity (e.g., Myin-Germeys et al., 2018).

Finally, the global temporal context in which this study took place warrants mention. All participants completed this study when their daily lives were likely affected by the COVID-19 pandemic. Because participants took the screener survey after pandemic-related social distancing measures were in place, our sample reflects participants who were still engaging in sexual activity despite these restrictions. While we cannot comment on the potential effects of pandemic-related events on people’s willingness to engage in partnered sexual activity, our study design systematically controlled for the turbulent week-to-week variability in daily life during this time by having all participants complete the present study during the same 28-day period.

### Conclusion

In the present study, we provided evidence that within-person variability can explain a substantial proportion of variance in internal and external sexual consent – at least 50% and up to 80%. Our findings support Willis and Jozkowski’s (2019) emphasis on the nuanced nature of sexual consent, which they defined as a “willingness to engage in a particular behavior with a particular person within a particular context” (p. 1723). Indeed, people’s sexual consent seems to vary greatly based on the context in which partnered sexual events occur. Going forward, experience sampling and similar methodologies should be employed to better understand the time-varying contextual factors relevant to sexual consent.

## Supplementary Material

Supplemental Material
