# Supplementary material for: Assessing the Within-Person Variability of Internal and External Sexual Consent
Source: J Sex Res. 2021 Apr 30;58(9):1173–83. doi: 10.1080/00224499.2021.1913567 (PMC9239691; doi:10.1080/00224499.2021.1913567)
Supplement: Supplemental Material [file HJSR_A_1913567_SM7755.doc]

Online Supplementary Material

Supplementary Figure 1

*Post hoc power analysis was conducted using the ‘EMAtools’ package (Kleiman, 2017). This curve depicts level of power (1 – β = .8) for experience sampling study designs that include 113 participants, a 28-day study period, three time points per day, and an estimated ICC of .5. Vertical lines indicate hypothetical completion rates.*


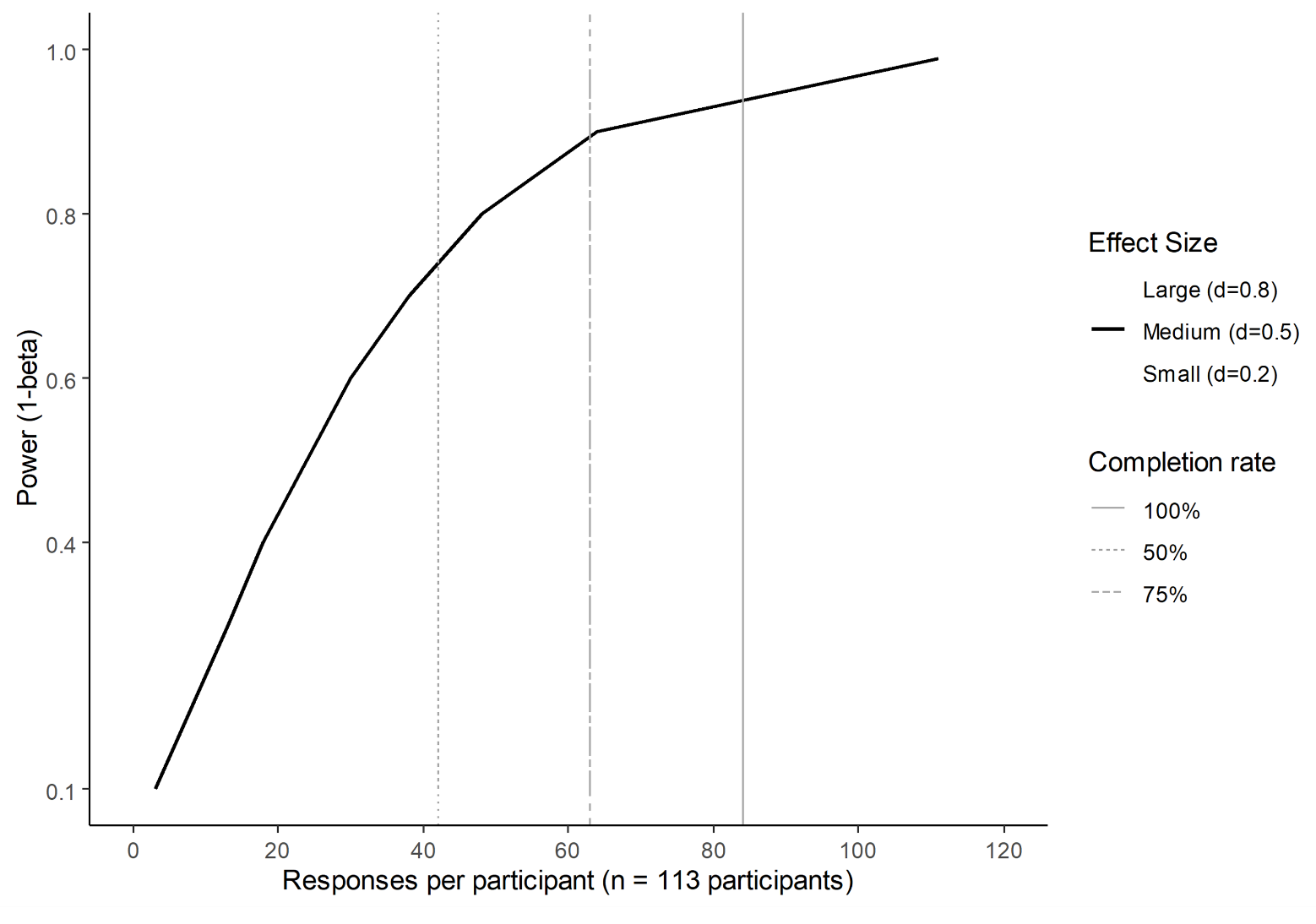


Supplementary Table 1

*Event-Level Descriptive Statistics and Correlations (N = 1189)*

|  | *M* | *SD* | Range | Skew. | Kurt. | 1. | 2. | 3. | 4. | 5. | 6. | 7. | 8. |
| --- | --- | --- | --- | --- | --- | --- | --- | --- | --- | --- | --- | --- | --- |
| 1. Physical response | 8.18 | 2.36 | 0 – 10 | -1.49 | 1.58 | — |  |  |  |  |  |  |  |
| 2. Safety/comfort | 8.96 | 1.50 | 1 – 10 | -1.92 | 4.29 | .39*** | — |  |  |  |  |  |  |
| 3. Arousal | 8.45 | 1.95 | 0 – 10 | -1.55 | 2.48 | .72*** | .49*** | — |  |  |  |  |  |
| 4. Agreement/want | 9.28 | 1.30 | 2 – 10 | -2.30 | 6.20 | .37*** | .52*** | .49*** | — |  |  |  |  |
| 5. Readiness | 8.66 | 1.80 | 0 – 10 | -1.70 | 2.99 | .50*** | .60*** | .59*** | .58*** | — |  |  |  |
| 6. Explicit cues | 7.84 | 2.64 | 0 – 10 | -1.42 | 1.23 | .26*** | .27*** | .27*** | .29*** | .35*** | — |  |  |
| 7. Implicit cues | 6.01 | 3.20 | 0 – 10 | -0.48 | -1.01 | .02 | .06 | .03 | .01 | .06* | -.16*** | — |  |
| 8. Verbal cues | 6.69 | 3.42 | 0 – 10 | -0.81 | -0.76 | .16*** | .08 | .13*** | .11*** | .20*** | .50*** | -.10** | — |
| 9. Nonverbal cues | 6.94 | 3.05 | 0 – 10 | -0.93 | -0.27 | .06* | .11*** | .11*** | .15*** | .13*** | -.07* | .56*** | -.30*** |

*Note.* Descriptive statistics and correlations were calculated for the 1189 semi-random assessments for which participants reported a partnered sexual event.

**p* < .05. ***p* < .01. ****p* < .001.

Supplementary Table 2

*Person-Level Descriptive Statistics and Correlations (N = 113)*

|  | *M* | *SD* | Range | Skew. | Kurt. | 1. | 2. | 3. | 4. | 5. | 6. | 7. | 8. |
| --- | --- | --- | --- | --- | --- | --- | --- | --- | --- | --- | --- | --- | --- |
| 1. Physical response | 8.39 | 1.43 | 3 – 10 | -1.07 | 1.41 | — |  |  |  |  |  |  |  |
| 2. Safety/comfort | 9.00 | 1.09 | 4.7 – 10 | -1.47 | 2.35 | .57*** | — |  |  |  |  |  |  |
| 3. Arousal | 8.61 | 1.25 | 4.5 – 10 | -1.03 | 0.72 | .86*** | .73*** | — |  |  |  |  |  |
| 4. Agreement/want | 9.38 | 0.84 | 6 – 10 | -1.63 | 2.46 | .51*** | .72*** | .63*** | — |  |  |  |  |
| 5. Readiness | 8.78 | 1.16 | 4.7 – 10 | -0.94 | -0.59 | .70*** | .83*** | .76*** | .73*** | — |  |  |  |
| 6. Explicit cues | 8.06 | 1.39 | 4.2 – 10 | -0.42 | -0.51 | .45*** | .44*** | .49** | .43*** | .56*** | — |  |  |
| 7. Implicit cues | 6.03 | 2.31 | 0 – 10 | -0.43 | -0.43 | -.02 | .07 | -.02 | .02 | .09 | -.08 | — |  |
| 8. Verbal cues | 6.85 | 2.03 | 0 – 10 | -0.72 | 0.47 | .09 | .01 | .06 | .04 | .13 | .42*** | -.02 | — |
| 9. Nonverbal cues | 6.89 | 2.28 | 0.3 – 10 | -0.83 | 0.18 | .06 | .17 | .09 | .25** | .21* | .06 | .71*** | -.25** |

*Note.* Descriptive statistics and correlations were calculated using participants’ average scores from the 28-day study period.

**p* < .05. ***p* < .01. ****p* < .001.

Supplementary Table 3

*Fixed Effects for the Full Multilevel Models Including Person-Level Sociodemographic Variables as Predictors (N = 112)*

|  | Internal Sexual Consent | | | |  | External Sexual Consent | | | |
| --- | --- | --- | --- | --- | --- | --- | --- | --- | --- |
|  | *β* | SE | 95% CI | *p* |  | *β* | SE | 95% CI | *p* |
| Intercept | 7.68*** | .55 | [7.33, 7.99] | <.001 |  | 4.97*** | .68 | [3.63, 6.32] | <.001 |
| Gender | -.15 | .18 | [-.49, .20] | .401 |  | -.24 | .23 | [-.69, .20] | .286 |
| Age | .00 | .02 | [-.04, .04] | .952 |  | -.00 | .03 | [-.05, .04] | .849 |
| Race/ethnicity | -.13 | .19 | [-.51, .25] | .512 |  | -.18 | .25 | [-.66, .31] | .476 |
| Relationship length | .02 | .02 | [-.03, .06] | .483 |  | .02 | .03 | [-.04, .07] | .553 |
| Type of sexual behavior | .47*** | .05 | [.37, .56] | <.001 |  | .31*** | .08 | [.14, .47] | <.001 |
| Internal sexual consent | — | — | — | — |  | .12* | .06 | [.01, .23] | .041 |

*Note*. **p* < .05. ****p* < .001.

Supplementary Table 4

*Fixed Effects for the Post Hoc Multilevel Models with Internal Consent Feelings Predicting Individual Consent Cues (N = 113)*

|  | Explicit Consent Cues | | | |  | Implicit Consent Cues | | | |
| --- | --- | --- | --- | --- | --- | --- | --- | --- | --- |
|  | *β* | SE | 95% CI | *p* |  | *β* | SE | 95% CI | *p* |
| Intercept | 7.24*** | .24 | [6.77, 7.72] | <.001 |  | 6.12*** | .31 | [5.50, 6.73] | <.001 |
| Type of sexual behavior | .29*** | .08 | [.14, .45] | <.001 |  | -.03 | .09 | [-.20, .15] | .756 |
| Internal sexual consent | .61*** | .07 | [.48, .75] | <.001 |  | .31*** | .08 | [.16, .46] | <.001 |
|  | Verbal Consent Cues | | | |  | Nonverbal Consent Cues | | | |
| Intercept | 5.28*** | .32 | [4.64, 5.91] | <.001 |  | 7.28*** | .30 | [6.69, 7.88] | <.001 |
| Type of sexual behavior | .60*** | .10 | [.39, .80] | <.001 |  | -.14 | .09 | [-.32, .03] | .104 |
| Internal sexual consent | .53*** | .09 | [.35, .71] | <.001 |  | .45*** | .08 | [.30, .60] | <.001 |

*Note*. ****p* < .001.
